# The effect of sexually transmitted disease education via instagram on the knowledge and attitudes of university students: a pilot study

**DOI:** 10.1590/1806-9282.20241602

**Published:** 2025-05-02

**Authors:** Özlem Can Gürkan, Begüm Kırık, Yusuf Eş, Necmedin Modanlar, Enes Atakan, Furkan Demir

**Affiliations:** 1 Marmara University, Faculty of Health Sciences, Department of Nursing – İstanbul, Türkiye.; 2 Yeditepe University, Faculty of Health Sciences, Department of Nursing – İstanbul, Türkiye.

**Keywords:** Social media, Training, STDs

## Abstract

**OBJECTIVE:**

The aim of this study was to investigate the effects of sexually transmitted disease education provided via Instagram on university students’ knowledge and attitudes about sexually transmitted diseases.

**METHODS:**

This pretest-posttest control group experimental study was conducted on 69 participants (Instagram group: 30, control group: 39). Participants in the Instagram group followed the @sexualinfectmarmara Instagram account prepared by the researchers. Five modules about sexually transmitted diseases prepared by the researchers were shared on this account.

**RESULTS:**

When the pre-training STD scores of the Instagram and control groups were compared with the analysis of covariance (ANCOVA) analysis, it was found that the scores of the Instagram group were statistically significantly higher than those of the control group. No significant difference was found between the sexually transmitted disease scores. After the training, the participants in the Instagram group responded significantly more with disagreement to the statement "sexually transmitted disease patients are easily recognized in society."

**CONCLUSION:**

Instagram can be a new and alternative educational tool for increasing the sexually transmitted disease knowledge and attitude levels of university students.

## INTRODUCTION

Sexually transmitted diseases (STDs) are increasing globally and are a problem that particularly affects young adults^1^. Due to social and biological factors and engaging in risky sexual behaviors due to peer and social media influences, young adults are at a high risk of acquiring STDs. In this regard, the most important problem is that young people do not have sufficient knowledge about STDs^2^. With the development of technology, websites, mobile applications, and social media tools are used to provide information about STDs. It has been reported that these methods can be effective in reducing STDs^3,4^. A large proportion of young people are Internet and social media users, which provides an important opportunity for them to learn about STDs^5^.

Today, various technological tools such as websites and mobile applications are used to provide STD education to young people. However, there are no studies on STD education via Instagram. Instagram has 1 billion users worldwide and is a popular social media platform where photos and videos are shared. It has been stated in the literature that Instagram is used as a low-cost and effective tool for providing education on various diseases^6,7^.

This study was conducted to evaluate the effect of education provided via Instagram on the knowledge and attitude levels of university students about some of the common STDs, namely chlamydia, human papillomavirus (HPV), human immunodeficiency virus (HIV), and syphilis.

## RESEARCH HYPOTHESES

H1: After the training, the Instagram group will have higher STD Knowledge Test (STDKT) scores than the control group.

H2: After the training, the Instagram group will have higher STD (chlamydia, HPV, HIV, and syphilis)-Specific Knowledge Test (STDSKT) scores than the control group.

## METHODS

### Study design and setting

The present study is an experimental study conducted with a pretest-posttest control group. The study was conducted at the Faculty of Health Sciences, Marmara University, between January 2022 and February 2023.

### Participants

The study included students between the ages of 18 and 25 years, studying in the Physiotherapy and Rehabilitation, Health Management, and Nutrition-Dietetics departments, who were in their second year, used Instagram, had not received training on STDs, had not been diagnosed with STDs, and agreed to participate in the study. Students studying in the Physiotherapy and Rehabilitation (n=8) and Nutrition-Dietetics departments formed the Instagram group (n=22), and students studying in the Health Management department (n=39) formed the control group. The allocation of students to the two groups based on departments was decided by drawing lots.

### Sample size calculation

The sample size was calculated using OpenEpi, version 3. With a confidence interval of 95% and a power of 80%, the Instagram group post-education STDKT score was found to be 28.50±2.01 and the control group post-education STDKT score was found to be 26±4.1. Based on the calculation made, it was decided that at least 27 people should be included in each group. Considering that there may be a loss of cases in each group, 90 participants (Instagram group: 45, control group: 45) were included in the study. Twenty-one participants withdrew from the study/were excluded from the study. Finally, the study was conducted on 69 participants (Instagram group: 30, control group: 39).

### Data collection tools and data acquisition

Data were obtained using the Demographic Information Form (DIF), sexually transmitted disease Knowledge Test (STDKT), sexually transmitted disease Specific Knowledge Test (STDSKT), and STD Attitude Survey (STDAS), before and after the training.

- DIF: This form consists of a total of seven questions that evaluate the sociodemographic characteristics of the participants (age, gender, education level of mother and father, source from where they get information about health problems, etc.).
- STDKT: This test aims to evaluate the general knowledge level of individuals about STDs^8^. In our study, this knowledge test was employed with a total of 20 questions (items that were not compatible with the educational content were removed), and each correct answer was awarded 5 points. A maximum score of 5–100 can be rated for the knowledge level of individuals about STDs.. The Kuder–Richardson (KR)-21 reliability coefficient, which was reported as 0.82 in the study of Siyez and Siyez^8^, was found to be 0.80 in our study.
- STDSKT: This test includes 10 questions on chlamydia, HPV, HIV, and syphilis. The purpose of the test is to measure the level of knowledge about the agents, symptoms, findings, treatment methods, and possible situations that may occur if these diseases are not treated^9^. Each correct answer is worth 10 points. According to the difficulty index evaluation of the test, four questions were classified as easy (40%), two questions as medium (20%), and four questions as difficult (30%). As a result of the discrimination index of the test, six items were found to be excellent, two items were acceptable, and two items were found to be weak. The KR-20 reliability coefficient of the test was determined as 0.60 in our study.
- STDAS: This three-question survey, prepared in line with the relevant literature, aims to measure the basic attitudes of the participants about STDs^10,11^.

### Sexually transmitted disease education program

Five modules about STDs prepared by the researchers were used in the education program, of which the general information module about STDs includes general information about the frequency of STDs and their types, signs and symptoms, treatment options, complications, and protection methods. The other four modules provide information about the causative agents, transmission routes, diagnostic tests, treatment modalities, and protection methods of HPV, syphilis, HIV/AIDS, and chlamydia diseases. The information in the modules was prepared in the form of descriptive posts and animation shows (a total of 28 contents) and was shared on an Instagram account called @sexualinfectmarmara (https://www.instagram.com/sexualinfectmarmara?utm_source=qr&igsh=YXJwODVyNTU1cmln).

### Data collection

The researchers invited second-year students for participation from the departments where they would conduct the study by explaining the purpose of the study. Students who were willing to participate were asked to sign an informed consent form, and their contact information was obtained.

### Instagram group

A pre-test data collection link, which was transferred to Google Drive, was sent to the participants via WhatsApp. Participants who filled out the pre-test forms were asked to follow the @sexualinfectmarmara Instagram account. After a sufficient number of participants were attained, the STD training modules were shared on Instagram every day for 4 weeks. Two weeks after the final content was shared, a post-test data collection link was sent to the participants via WhatsApp.

### Analysis of data

Categorical data were analyzed using the χ^2^ test and Bonferroni correction test. The Kolmogorov-Smirnov test was used to check the data for normal distribution. Parametric data were evaluated using the independent samples t-test and paired sample t-test. The analysis of covariance (ANCOVA) test was applied, assuming the pre-education scores as covariates and the post-education scores as dependent variables. It was evaluated at a p<0.05 significance level.

### Ethical aspects of the research

Ethics committee approval was obtained from the Non-Interventional Clinical Research Ethics Committee, Faculty of Health Sciences, Marmara University (date: 30.12.2021/protocol no. 124). Permission for the use of STDKT was received from Siyez via e-mail.

## RESULTS

Data on the characteristics of the participants in the study are presented in Table 1. There was no statistically significant difference between the groups in terms of age, gender, and maternal education level (p>0.05) (Table 1). It was determined that 90.00% (n=27) of the participants in the Instagram group had used social media/Internet to obtain information about STDs in the past, and there was a statistically significant difference between the groups (p<0.05).

**Table 1:** Comparison of the distribution of characteristics of participants in the groups.

Characteristics		Instagram group (n=30)		Control group (n=39)		t	p
		X+SD		X+SD			
Age (years)		20.50±0.97		20.07±0.89		1.86	0.06
		N	%	N	%	χ^2^	p
Gender							
	Female	25	83.3	29	74.4	0.80	0.37
	Male	5	16.7	10	25.6		
Mother's education level							
	Primary school	12	40.00	5	12.80	7.63	0.054
	Secondary school	7	23.30	12	30.80		
	High school	10	33.30	17	43.60		
	University	1	3.30	5	12.80		
Father's education level							
	Primary school	8	26.70	1	2.60	11.80	**0.008** *
	Secondary school	8	26.70	6	15.40		
	High school	8	26.70	18	46.20		
	University	6	20.00	14	35.90		
Total		30	100.00	39	100.00		
From where they have learned about STDs in the past**							
	Social media—internet	27	90.00	22	56.40	9.29	**0.002** *
	Friends—peers	14	46.70	26	66.70	2.78	0.09
	Family	5	16.70	17	43.60	5.65	**0.01** *

* p<0.05.

** Row percentage is used.

STD: sexually transmitted disease. Statistically significant values are denoted in bold. X+SD: represents mean±standard deviation.

It was found that both the pre-training STDKT scores (p<0.05 and p<0.001, respectively) and the post-training STDSKT scores (p<0.001 and p<0.001, respectively) of the participants in the Instagram and control groups were statistically significantly different (Table 2).

**Table 2:** Comparison of the scores of the participants in the groups from the sexually transmitted disease Knowledge Test and the sexually transmitted disease-specific Knowledge Test.

"Results for knowledge tests	Pre-Training			Post-training					
	Group	X±SD	t; p^1^	X±SD	t; p^2^	t; p^3^	F	ηp^2^	p^4^
STDKT	Instagram group (n=30)	56.16±25.14	2.65; 0.01*	68.83±19.50	6.08; 0.00**	−2.77; 0.01*	25.63	0.28	0.00*
	Control group (n=39)	42.23 ±15.84		42.69±16.17		−0.19; 0.84			
STDSKT	Instagram group (n=30) Control group (n=39)	46.66±16.88 25.12±11.89	6.21; 0.00**	52.66±23.6230.00±16.54	4.47; 0.00**	−1.96; 0.05 −1.65; 0.10	1.97	0.029	1.65

p^1^: Comparison of pre-training knowledge test scores between groups (independent samples t-test). p^2^: Comparison of post-training knowledge test scores between groups (independent samples t-test). p^3^: Comparison of pre-training and post-training knowledge test scores within the group (paired sample t-test). p^4^: ANCOVA.

* p<0.05;

** p<0.001.

STDKT: sexually transmitted disease Knowledge Test; STDSKT: sexually transmitted disease Specific Knowledge Test.

It was also found that the post-training STDKT score of the Instagram group increased statistically significantly compared to the pre-training level (p<0.001), while the pre-training STDSKT scores did not change after the training (p=0.05) (Table 2).

When the post-training STDKT scores of the Instagram and control groups were compared with the ANCOVA analysis, assuming the pre-training scores as covariates, it was found that the scores of the Instagram group increased statistically significantly compared to the control group (p<0.001). When the post-training STDSKT scores of the Instagram and control groups were compared with the ANCOVA test, it was determined that the scores of both groups were similar (p>0.05) (Table 2).

While the responses to the statement "patients with STDs are easily recognized in society" were similar between the groups before the training, it was found that there was a statistically significant difference between the groups after the training, and this difference was due to those who disagreed (p<0.05) (Table 3).

**Table 3:** Comparison of the distribution of responses to sexually transmitted diseases given by participants in the groups before and after the training.

Expressions		Before training						After training					
		Experimental group (n=30)		Control group (n=39)				Experimental group (n=30)		Control group (n=39)			
		n	%	N	%	χ^2^	p	N	%	n	%	χ^2^	P
Teenagers are more vulnerable to STDs													
	Agree	23	76.7	31	79.5	0.77	0.5	24	80	29	74.4	2.41	0.29
	Disagree	–	–	–	–			6	20	7	17.9		
	Undecided	7	23.3	8	20.5			–	–	3	7.7		
Patients with STDs are easily recognized in society													
	Agree	9	30	18	46.2	3.41	0.18	7	23.30	16	41.00	8.62	0.01*
	Disagree	13	43.3	9	23.1			12	40.00	4	10.30		
	Undecided	8	26.7	12	30.8			11	36.70	19	48.70		
Protecting against STDs is difficult and costly													
	Agree	11	36.7	21	53.8	5.31	0.07	11	36.7	22	56.4	2.79	0.24
	Disagree	13	43.3	7	17.9			10	33.3	10	25.6		
	Undecided	6	20	11	28.2			9	30	7	17.9		
Total		30	100	39	100			30	100	39	100		

Chi-square test; Bonferroni correction test.

* p<0.05.

STD: sexually transmitted disease.

## DISCUSSION

This study was conducted to evaluate the effect of education provided via Instagram on the knowledge and attitude levels of university students about some common STDs. The findings show that hypothesis H1 is supported. It was determined that the STD scores of the Instagram group increased significantly after the education compared to the control group. This situation emphasizes the potential of social media-based education to increase the general knowledge level of the participants. It is thought that platforms such as Instagram can be an effective educational tool, especially for young people and individuals with high digital media usage^10^.

However, the results of hypothesis H2 did not occur as expected. No statistically significant difference was found between the STDSKT scores of the Instagram and control groups after the training (p>0.05). This result shows that the potential of education provided through social media to increase knowledge on certain topics may be limited. It supports the idea that topics requiring special knowledge such as chlamydia, HPV, HIV, and syphilis should be addressed with a more in-depth and interactive training method. It is understood that social media training can be effective on basic information, but more comprehensive methods should be adopted for special information^12^. A similar 2022 study assessed the knowledge of students in public high schools in poor communities about HPV and STDs, their attitudes toward these diseases, and their attitudes toward the prevention of these diseases and concluded that adolescents had very limited knowledge about HPV and cervical cancer^13^.

The significant difference in the responses to the statement "STD patients are easily recognized in society" after the training reveals the potential of social media training to change the social perceptions of the participants. It was observed that the participants had a more critical perspective after the training. This indicates the potential of training conducted via social media to provide participants with a thought-provoking perspective on issues, such as stigmatization and social perception.

### Limitations of the study

The study findings cannot be generalized to all university students. Since there are no STDSKT and STDAS that have been examined for validity and reliability in our country, forms prepared by the researchers were used. Another limitation is the use of objectively analyzed quantitative questionnaires, which relied on the self-report of students on their knowledge about the subjects without any confirmation. As a result, the knowledge assessed is subjective or perceived. Moreover, these questionnaires do not provide an opportunity to explore socioeconomic factors such as religious beliefs or to assess the prevalence of sexually transmitted infections/HPV and vaccination status. Therefore, further studies are required.

## CONCLUSION AND RECOMMENDATIONS

This study highlights the role of social media and the Internet in education and shows that platforms such as Instagram can be an important tool for acquiring knowledge. However, it should be noted that traditional education methods are also effective for gaining more in-depth knowledge on certain topics. Future research should examine the long-term effects of social media-based education programs and how they affect participants’ social perceptions in more detail. It is also thought that the content and presentation of social media education should be carefully designed to maximize their potential to increase participants’ knowledge.
